# IRF8 Is an AML-Specific Susceptibility Factor That Regulates Signaling Pathways and Proliferation of AML Cells

**DOI:** 10.3390/cancers13040764

**Published:** 2021-02-12

**Authors:** Franziska Liss, Miriam Frech, Ying Wang, Gavin Giel, Sabrina Fischer, Clara Simon, Lisa Marie Weber, Andrea Nist, Thorsten Stiewe, Andreas Neubauer, Andreas Burchert, Robert Liefke

**Affiliations:** 1 Institute of Molecular Biology and Tumor Research (IMT), Philipps University of Marburg, 35043 Marburg, Germany; franziska.liss@uni-wuerzburg.de (F.L.); fische4t@staff.uni-marburg.de (S.F.); clara.simon@imt.uni-marburg.de (C.S.); lisa.weber@imt.uni-marburg.de (L.M.W.); 2 Clinic for Hematology, Oncology, Immunology and Center for Tumor Biology and Immunology, Philipps University of Marburg, 35037 Marburg, Germany; frechm@staff.uni-marburg.de (M.F.); wangyin2@staff.uni-marburg.de (Y.W.); giel@staff.uni-marburg.de (G.G.); neubauer@staff.uni-marburg.de (A.N.); burchert@staff.uni-marburg.de (A.B.); 3 Genomics Core Facility, Institute of Molecular Oncology, Member of the German Center for Lung Research (DZL), Philipps University of Marburg, 35043 Marburg, Germany; andrea.nist@imt.uni-marburg.de (A.N.); thorsten.stiewe@uni-marburg.de (T.S.)

**Keywords:** acute myeloid leukemia, IRF8, transcription, signaling, proliferation, CRISPR, ChIP-Seq

## Abstract

**Simple Summary:**

Despite progress, acute myeloid leukemia (AML) remains one of the deadliest cancer diseases. The identification of novel molecular targets may allow developing innovative and alternative treatment options for AML. Using public data from genome-edited cancer cells, we identified factors that are specifically essential for AML cell growth. We validated the critical role of the transcription factor IRF8 and demonstrated that it modulates the function of the cells by regulating important signaling molecules. These results support that IRF8 may be a suitable molecular target for the treatment of AML.

**Abstract:**

Personalized treatment of acute myeloid leukemia (AML) that target individual aberrations strongly improved the survival of AML patients. However, AML is still one of the most lethal cancer diseases of the 21st century, demonstrating the need to find novel drug targets and to explore alternative treatment strategies. Upon investigation of public perturbation data, we identified the transcription factor IRF8 as a novel AML-specific susceptibility gene in humans. IRF8 is upregulated in a subset of AML cells and its deletion leads to impaired proliferation in those cells. Consistently, high IRF8 expression is associated with poorer patients’ prognoses. Combining gene expression changes upon IRF8 deletion and the genome-wide localization of IRF8 in the AML cell line MV4-11, we demonstrate that IRF8 directly regulates key signaling molecules, such as the kinases SRC and FAK, the transcription factors RUNX1 and IRF5, and the cell cycle regulator Cyclin D1. IRF8 loss impairs AML-driving signaling pathways, including the WNT, Chemokine, and VEGF signaling pathways. Additionally, many members of the focal adhesion pathway showed reduced expression, providing a putative link between high IRF8 expression and poor prognosis. Thus, this study suggests that IRF8 could serve as a biomarker and potential molecular target in a subset of human AMLs.

## 1. Introduction

Acute myeloid leukemia (AML) is a genetically heterogeneous myeloid disease caused by the hyperproliferation of undifferentiated myeloid progenitor cells [1]. AML is the most frequent acute leukemia in adults that typically progresses quickly and, if untreated, will lead to the patient’s death within weeks or months. Recent advances in understanding the pathogenesis and therapy of AML have improved the clinical outcomes of certain subtypes of AML such as FLT3-ITD-positive AML, but the five-year survival rate is still only at about 30–40% [1]. For some AMLs, such as those with complex karyotypes or TP53-pertubations, the prognoses remain persistently bad [2].

The conventional AML treatment via chemotherapy inhibits DNA synthesis, cell division, and mitosis, which affects both cancerous and normal cells, leading to side effects, including bone marrow suppression. Analyses for mutations in genes and their combination can offer a prognostic tool and help to create personalized treatment plans [3]. Molecular lesions in AML cells occur mainly at two distinct levels: The activation of cytoplasmic tyrosine kinases (FLT3; KIT) or signaling modulators such as NRAS and secondary lesions that alter the function of certain transcription factors or epigenetic regulators, such as CEBPα, RUNX1, RUNX1T, RAR and KMT2A [1,4]. Identifying and understanding these genetic alterations allows the targeting of specific cellular pathways, such as the FLT3 kinase pathway, which is impaired in about 30% of all AML cases [5]. However, despite advancements in the personalization of the AML treatment, many challenges remain. The common relapse of AML after first remission is most problematic [1].

Transcription factors are often mis-regulated in cancer, but they just recently emerged as potential drug targets in AML. In contrast to enzymes, which can be inhibited by targeting the active center, transcription factors were considered unsuitable as potential drug targets in the past. However, technical progress led to the discovery of novel drugs that can impair the function of transcription factors, allowing to utilize alternative treatment options [6,7]. The identification and characterization of transcription factors that are critical for AML progression could initiate the development of alternative drugs that target specific gene regulatory pathways in AML. Drugs like these could pave the path for an improved survival of AML patients. Here we surveyed publicly available perturbation data and identified several candidates as novel AML-specific susceptibility genes. For further investigations, we chose the transcription factor IRF8 (interferon regulatory factor 8, previously named ICSBP).

IRF8 belongs to the transcription factor family of interferon regulatory factors (IRFs) that play a variety of roles in the hematopoietic system and are involved in the type I interferon response [8]. IRF8 is best known for its function in the development and maturation of myeloid cells [9]. It regulates lineage-committed progenitors to promote monocyte maturation and to suppress neutrophil production [10,11]. IRF8 has previously been described to function as a tumor suppressor in multiple cancer types [12,13], including chronic myeloid leukemia (CML) [14,15,16,17] and murine acute promyelocytic leukemia (APL), a subclass of AML [18,19]. In contrast, our investigations suggest a pro-proliferative role of IRF8 in AML cell lines and connects high IRF8 expression with poorer patient outcome. This indicates a potential oncogenic function of IRF8 in the context of human AML. In line with this, IRF8 has recently emerged as a potential therapeutic marker in AML, especially in the context of treatment with the RARα agonist tamibarotene (SY-1425) and hypomethylating agents [20]. IRF8 has also been described to be regulated through a super-enhancer in a subset of AML cell lines and patients, which drives gene expression of AML-associated transcription factors [21,22]. Thus, IRF8 may act distinctly in AML compared to other cancer types, which warrants further investigation. Importantly, a peptide inhibitor for the related protein IRF5 has recently been described [23], suggesting that the members of the IRF protein family are potentially suitable for chemical inhibition [24].

To validate a critical role of IRF8 in AML, we studied its influence on cellular properties, its genomic targets, and its role in gene regulation in the AML cell line MV4-11. Our work suggests that IRF8 may be a promising molecular target in AML.

## 2. Materials and Methods

### 2.1. Cell Culture

HEK-293-T cells were cultured in DMEM/F-12, GlutaMAX™ Supplement (Gibco, 31331028) supplemented with 10% fetal bovine serum (Sigma, F7524) and 1% Penicillin/Streptomycin (Sigma Aldrich). MV4-11, MOLM13, HL-60, THP1, and NB4 cells were cultured in RPMI 1640 Medium, GlutaMAX™ Supplement (Gibco, 61870010) supplemented with 15% fetal bovine serum (Sigma, F7524) and 1% Penicillin/Streptomycin (Sigma Aldrich). Cell growth was assessed by seeding 0.15 × 10^6^ cells in triplicates in 500 µL medium on 24-well plates and counting of cells in three-day intervals. Cells were stained with trypan blue and counted using a Neubauer counting chamber.

### 2.2. Cell Extraction

Whole cell extract was prepared with RIPA-Buffer (25 mM Tris/HCL pH 7.5, 150 mM NaCl, 1% NP-40, 0.5% Sodium deoxycholate, 0.1% SDS; 0.5 mM PMSF and 1 × Protease Inhibitor Cocktail (PIC) were added directly before use). The protein concentration was measured with the “DC™ Protein Assay” (Bio Rad) at 750 nm and estimated by a standard BSA curve.

### 2.3. Cellular Fractionation

Fractionation assays were performed to determine the intracellular localization of IRF8 using the “Subcellular Protein Fractionation Kit for Cultured Cells” by Thermo Fisher (78840) according to the manufacturer’s instructions. Six-well plates were used corresponding to a packed cell volume of 20 µL. The same volumes of all fractions of interest were loaded on SDS-PAGE gels.

### 2.4. IRF8 Knockout/Knockdown

To generate stable knock-out cell lines, MV4-11 were subjected to lentiviral infection. Virus was produced in HEK-293-T by transfection with the packaging plasmids pMD2.g, psPAX2 and lentiCRISPRv2 (Addgene #52961) [25], containing gRNA directed against *IRF8*. Standard FuGENE transfection was used. Supernatant containing virus was collected 48h after transfection.

AML cell lines were lentivirally infected and selected with 1µg/mL of puromycin 36h after infection. MV4-11 knockout clones were generated by limited dilution, analyzed for IRF8 expression on protein and mRNA level, and clones were genotyped by PCR of the targeted genomic locus and Sanger sequencing. MV4-11 IRF8 ko clone 1 and 2 were generated by gRNA1 (GCGTAACCTCGTCTTCCAAG) and clone 3 by gRNA2 (GCTTGCCAGCGTGTTTCCAA).

### 2.5. Antibodies

The following antibodies were used: IRF8 (Santa Cruz, CA, USA, sc-365042), β-actin (Santa Cruz, CA, USA, sc-47778), H2B (Millipore, Burlington, MA, USA, 07-371), SP1 (self-made, [26]), Tubulin (Millipore, Burlington, MA, USA, MAB3408), GAPDH (14C10, Cell Signaling, Danvers, MA, USA, #2118)

### 2.6. Cell Cycle Analysis

Cells were harvested and washed twice in 1 × PBS and 1% FBS (washing buffer). Cells were fixed by resuspension in 1 mL washing buffer and slow addition of 4 mL 100% ice-cold ethanol whilst vortexing.

The cells were rehydrated by washing twice in 1 mL washing buffer before staining. Then, they were resuspended in 1 mL staining buffer (1 × PBS + 1% FCS, 50 μg/mL PI, 100 μg/mL RNase) and incubated 30 min at RT in the dark. Samples were measured with a LSR II flow cytometer. Data were analyzed by FlowJo v10.6.1.

### 2.7. Pappenheim Staining

To assess the morphology of cells, they were subjected to a Pappenheim’s staining. MV4-11 cells were spun onto a glass slide by cytospin. 8 × 10^4^ to 1 × 10^5^ cells were resuspended in 150 µL PBS and centrifuged onto the slides at 500 g for 5 min. The slides were left to dry at 4 °C overnight. Cells were stained in 50% May-Grünwald solution for 7 min and washed in ddH2O until no more color was coming of the slides. Then, they were stained in 10% Giemsa for 25 min, washed again and left to dry at 4 °C. The water used was adjusted to a pH of 6.8 beforehand. Slides were stored long-term at 4 °C and assessed microscopely.

### 2.8. RT-qPCR

RNA was isolated using the RNeasy Mini kit (Qiagen, 74104) and transcribed into cDNA with the Tetro cDNA Synthesis Kit (Bioline BIO-65043). qPCR was performed with the ImmoMix reagent (Bioline, BIO-25020) and gene expression was normalized to GAPDH expression. RT-qPCR Primers are presented in Table S1.

### 2.9. Chromatin Immunoprecipitation

For chromatin preparation 5 × 10^5^ cells per mL were seeded on three 15 cm plates with 25 mL medium each and cultured for 48 h. To cross-link DNA and proteins, 1%-formaldehyde was added and incubated at room temperature for 10 min. To stop the reaction 125 mM glycine was added for 5 min. Cells were transferred to a 50 mL tube and centrifuged at 2000 rpm and 4 °C for 10 min. After the medium was aspirated cells were washed twice with 10 mL cooled 1 × PBS, thereby bringing together cells of the same cell line in a 50 mL falcon. In-between washing steps, cells were centrifuged at 2000 rpm and 4 °C for 10 min. The pellet was resuspended in 1 mL buffer B (10 mM EDTA, 0.5 mM EGTA, 10 mM HEPES/KOH (pH 6.5), 0.25% Triton X-100) per 15 cm plate, transferred to a 15 mL falcon and centrifuged at 2000 rpm and 4 °C for 5min. The supernatant was aspirated, and the pellet was resuspended in 1 mL buffer C (10 mM EDTA, 0.5 mM EGTA, 10 mM HEPES/KOH (pH 6.5), 200 mM NaCl) per 15cm plate. After 10 min incubation on ice, the suspension was centrifuged at 2000 rpm and 4 °C for 5 min, the supernatant aspirated and the pellet was resuspended in 200–250 µL buffer D (10 mM EDTA, 50 mM Tris-HCl (pH 8), 1% SDS, 1 × Protease Inhibitor Cocktail (PIC) by Roche), depending on the size of the pellet. After an incubation period of 10min on ice, the chromatin was sheered 2 × 6 min in a pre-cooled bioruptor, transferred to a 1.5 mL-tube and centrifuged at 13,000 rpm and 4 °C for 10 min. The concentration of the soluble chromatin fraction was measured at a Nano-Drop. The chromatin immunoprecipitation was performed with the “one day ChIP kit” (Diagenode, Denville, NJ, USA) and performed according to the manual. Per IP, 60 µL chromatin in the same concentration were used, adjusted to the lowest concentrated chromatin. Eluted DNA was either analyzed by ChIP-qPCR or analyzed by next generation sequencing. ChIP-qPCR primers are presented in Table S1.

### 2.10. Next Generation Sequencing

For ChIP-Seq, chromatin immunoprecipitated DNA was used for indexed sequencing library preparation using the Microplex library preparation kit v2 (Diagenode, Denville, NJ, USA). Libraries were purified on AMPure magnetic beads (Beckman). For RNA-Seq, total RNA was extracted from MV4-11 cell infected with control vector, or from IRF8 KO clones by using the RNeasy Mini system (Qiagen, Hilden, Germany) including an on-column DNaseI digestion. RNA integrity was assessed on an Experion StdSens RNA Chip (Bio-Rad, Budapest, Hungary). RNA-Seq libraries were prepared using the TruSeq Stranded mRNA Library Prep kit (Illumina, San Diego, CA, USA). RNA-Seq and ChIP-Seq libraries were quantified on a Bioanalyzer (Agilent Technologies, Santa Clara, CA, USA). Next-generation sequencing was performed on Illumina NextSeq 550.

### 2.11. Bioinformatical Analysis

ChIP-Seq data were aligned to the human genome using Bowtie [27]. Peak calling was performed with MACS2 with standard setting [28]. The overlap of IRF8 peaks from MV4-11 (this study) and THP1 [29] was performed using the Venn-Diagram tool within the Cistrome platform [30]. The genomic distribution of IRF8 was performed using CEAS tool in Cistrome. All IRF8 peaks overlapping with promoter sites (downloaded from the UCSC table browser) were considered as IRF8 promoter peaks. All others were non-promoter peaks. Heatmaps, binding profiles and bigwig files were created using DeepTools [31]. Enriched motifs were identified using HOMER [32], using all IRF8 peaks as input. Gene ontology analysis of IRF8 target loci was performed using GREAT [33]. ChIP-Seq tracks were visualized using the UCSC browser [34].

Following public ChIP-Seq datasets were used: IRF8 (THP1): GSM3514948 [29]; RNA Polymerase II: GSM2716705 [35]; H3K27me3: GSM1513828 [36]; H3K27ac: GSM1513830 [36]; H3K4me3: GSM1513832 [36]; H3K4me1: GSM1587892 [21]; ATAC-Seq: GSM2544216 [37]; PU.1: GSM624143 [38]; CTCF, GSM2544244 [37]; RUNX1: GSM2108052 [39]; FOS: GSM3112113 [40]; CEBPA: GSM3514946 [29].

RNA-Seq samples were aligned to the human transcriptome GENCODE v32 using RNA-Star (2.7.2b) [41]. Reads per gene were calculated using feature counts (2.0.1). Differentially regulated genes and normalized read counts were determined using DeSeq2 [42] (2.11.40.6). Genes with an at least 2-fold difference and a p-value below 0.01 were considered as differentially expressed genes. Gene set enrichment analysis (GSEA) [43] was performed with standard setting. KEGG pathway analysis was performed using the R package pathview [44].

### 2.12. Used Websites

Below the links to used databases and tools:

GEPIA: http://gepia.cancer-pku.cn/index.html
DepMAP: https://depmap.org/
GSEA: https://www.gsea-msigdb.org/
Bioconductor/R: https://www.bioconductor.org/
Galaxy: https://usegalaxy.org/
Xena Browser: https://xena.ucsc.edu/
UCSC Browser: https://genome.ucsc.edu/
GREAT: http://great.stanford.edu/
CCLE: https://portals.broadinstitute.org/ccle
CRISPR Screen Database: https://orcs.thebiogrid.org/

### 2.13. Statistical Analysis

Statistical comparisons of two groups was carried out using unpaired Student’s *t*-tests. Other P-values were determined by the used software/tool, such as GSEA [43], HOMER [32] and DeSeq2 [42].

## 3. Results

Previous work on AML have identified several important factors for the development of AML, such as the kinase FLT3, the transcription factor RUNX1 and the GTPase NRAS [4]. These factors were mostly identified via genetic screens that collected data about genetic alterations in cancer samples. However, apart from mutations or translocations, genetically unaltered, but overexpressed, factors may contribute to the progression of AML. The expansion of genome-wide data allows the discovery of so far undescribed factors, which could play critical roles in the context of acute myeloid leukemia. Such novel factors could be suitable for chemical targeting and may thus offer novel treatment options.

### 3.1. IRF8 Is a Susceptibility Gene Candidate in AML

**Figure 1:**
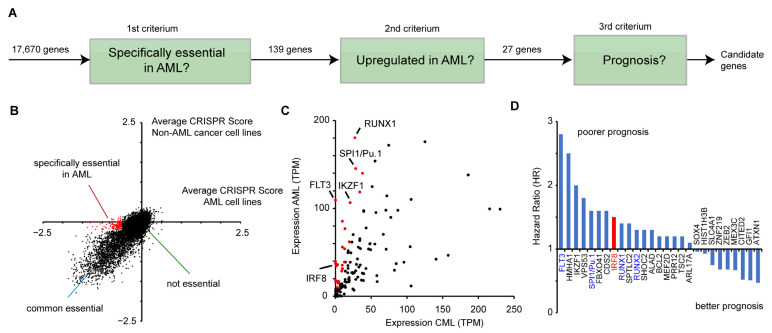
Identification of IRF8 as an acute myeloid leukemia (AML)-specific susceptibility gene. (**A**) Schematic representation of the selection process to identify potential candidates that play a role in AML. (**B**) Comparison of CRISPR scores of AML and non-AML cell lines [45]. Genes in red (*n* = 139), have an average CRISPR score below −0.5 in the AML cell lines, and a score above −0.2 in non-AML cell lines. See also Table S2. (**C**) Comparison of the gene expression of the 139 genes from (**B**) in AML and CML (“normal tissue”) samples, obtained from GEPIA. Red marked genes (*n* = 27) have an at least 3-fold increased expression in AML cells compared to the control. (**D**) Hazard ratio (HR) comparing the 25% highest and lowest expressing AML samples of the 27 selected genes from (**C**). Data for (**C**) and (**D**) were derived from GEPIA [46].

To identify potential novel drug targets in AML, we combined data from genome-wide perturbation data, cancer gene expression, and patient survival (Figure 1A). First, we made use of a recent CRISPR (Clustered Regularly Interspaced Short Palindromic Repeats) screen [45] that identified susceptible genes in a wide variety of human cancer cells. In total 17,670 genes were investigated on how their inactivation affects the proliferation of human cancer cell lines. A negative CRISPR score indicates that the gene deletion reduces the proliferation of the cells. To detect susceptible genes, that are specifically relevant in AML, we analyzed 13 AML cell lines (OCIAML5, OCIAML2, EOL1, OCIAML3, PL21, MONOMAC1, THP1, MV4-11, SKM1, MOLM13, P31FUJ, and NB4, TF1, see Table S3) in relation to other cancer cell lines. For this purpose, we compared the average CRISPR score from the AML cell lines with the average score of all other cancer cell lines (*n* = 345) (Figure 1B). For most genes, the two values are highly correlative, showing that their deletion impairs cell growth in AML cell lines and in the other cancer cell lines in a similar manner. However, several genes have a strong negative CRISPR score only in AML cell lines, but not in the other cancer cell lines, indicating that those genes are particularly important for AML cancer cell growth. For further analysis, we selected 139 genes that have an average CRISPR score below −0.5 in the AML cell lines, but higher than −0.2 in the other cancer cell lines (Figure 1B, Table S2).

Next, we investigated whether the selected genes show an aberrant expression in AML cell lines. Given that no normal tissue exists for AML, we compared the average expression levels of those genes in AML samples versus chronic myeloid leukemia (CML) cells (“normal tissue”), obtained from the GEPIA (Gene Expression Profiling Interactive Analysis) platform [46]. A fraction of the genes demonstrated a dramatically higher expression in AML, including key transcription factors, such as SPI1 (PU.1) and RUNX1 [47] (Figure 1C). Out of the 139 genes, we selected 27 highly upregulated genes (>3 fold increased expression, average TPM in AML samples >10) for further investigation (Figure 1C). Subsequently, we analyzed how their gene expression correlates with patient survival. For this purpose, we calculated the hazard ratio of high versus low expression of the respective genes, using the GEPIA platform, which utilizes data from the TCGA (The Cancer Genome Atlas) consortium. Of the 27 selected genes, we found that most of them have a hazard ratio higher than one, meaning that their high expression is correlative with a poorer prognosis (Figure 1D). Among these genes are well-known factors important for AML, including FLT3, SPI1, RUNX1, and RUNX2 [4,47], demonstrating that the chosen approach is suitable to identify susceptibility genes. Besides these already known factors involved in AML progression, we also identified several novel candidates, including the interferon regulatory factor 8 (IRF8, also ICSBP) (Figure 1D), which has been described as a tumor suppressor in several other cancer types before [12,13,14,15,16,17,18]. As we were able to identify a putative pro-proliferative and negative prognostic role of IRF8 in AML, we hypothesized that IRF8′s role in AML might vary from its function in other cancer types. As an inhibitor for the related protein IRF5 was recently described [23,24], IRF8 may also be a suitable drug target. Therefore, we decided to investigate the role of IRF8 in AML in more detail.

### 3.2. IRF8 Is Highly Expressed in Leukemia Cells and Its High Expression Is Linked to Poorer Survival

**Figure 2:**
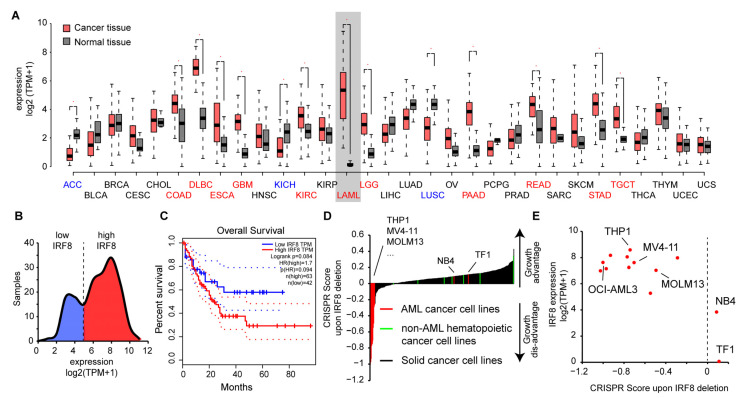
IRF8 is upregulated in AML and is associated with poor survival. (**A**) Gene expression of IRF8 in normal tissues (grey) and tumor samples (red). For LAML (Acute Myeloid Leukemia), samples from chronic myeloid leukemia (CML) cells are used as reference. Blue and red colored cancer names indicate significantly down- or upregulated IRF8 expression in those cancer types, respectively. Data from GEPIA [46]. (**B**) Distribution of IRF8 expression in TCGA AML cancer samples (*n* = 173). (**C**) Kaplan–Meier survival curve comparing AML patients with low (blue) and high (red) IRF8 expression. Data from GEPIA [46]. (**D**) CRISPR score in 350 human cancer cell lines. IRF8 deletion in most AML cell lines (in red) leads to a negative CRISPR score, demonstrating reduced proliferation after IRF8 deletion. Non-AML hematopoietic cancer cell lines (green) are shown in comparison. (**E**) Association between IRF8 expression and CRISPR score in human AML cell lines. See Table S3, for details about the cell lines.

To gain insights into the role of IRF8 in AML, we had a closer look at publicly available data. We observed that IRF8 is expressed in most tissue types at a high level, but with varying intensities. IRF8 is particularly strongly expressed in lung, intestinal, and blood tissues (Figure 2A), suggesting that IRF8 has additional functions besides its known role during blood cell development [9]. In several tissue types, such as in colon, pancreas, kidney, and stomach, we observed a significantly altered expression of IRF8 in cancer tissues compared to the corresponding normal tissues. In most of the cancer tissues, IRF8 get upregulated, while in some, such as in lung squamous cell carcinoma (LUSC) IRF8 is downregulated. Consistent with our hypothesis, the most dramatic difference between cancer and control samples can be noticed in AML. In this case, an about 400-fold increase in gene expression can be observed (Figure 2A), suggesting that AML cells strongly upregulate IRF8 during tumorigenesis. Interestingly, investigation of the TCGA data showed that AML samples can be subdivided into two classes based on their IRF8 expression. About 60% of the samples show a high IRF8 expression, while the other 40% show a lower expression (Figure 2B). In line with a potential oncogenic role of IRF8 in AML, we found that the TCGA samples with the high IRF8 expression are associated with a poorer prognosis (Figure 2C), suggesting that IRF8 overexpression in human AML is possibly disadvantageous. Due to the low number of samples in the TCGA dataset, further validation with larger patient numbers will however be important to confirm this conclusion.

To address the role of IRF8 in AML cells, we next investigated how IRF8 deletion influences the proliferation of AML cell lines in comparison to other cancer types using the above-mentioned CRISPR screen data [45]. In most non-AML cancer cells, including other hematopoietic cancer cells, IRF8 deletion is associated with a positive score, indicating that IRF8 deletion leads to an increased cell proliferation of the cells (Figure 2D). This result is consistent with the previously described function of IRF8 as a tumor suppressor [12,13]. However, for most AML cell lines, we found the opposite result, namely a strong negative score. Some AML cell lines, such as erythroleukemia cell line TF1 and the APL cell line NB4 cells, also have a positive CRISPR score, suggesting that not all AML cells depend on IRF8 expression. Further investigation revealed that those AML cells with a positive score also have the comparably lowest IRF8 expression (Figure 2E). These results suggest that AML cells with a high IRF8 expression do depend on the presence of IRF8 for proliferation, while cells with lowly expressed IRF8 may have adapted differently and do not require IRF8 for proliferation. Notably, the cell lines that are strongly dependent on IRF8 in the CRISPR screen belong to various AML subtypes (Table S3), indicating that the importance of IRF8 is not restricted to a certain AML class. Thus, AML could possibly be categorized into IRF8-dependent and IRF8-independent types.

Together, these results suggest that IRF8 upregulation in a subset of AML cells influences the properties of these cells, which in turn leads to a dependency on IRF8 for their proliferation.

### 3.3. IRF8 Deletion Impairs Growth and Properties of AML Cells

**Figure 3:**
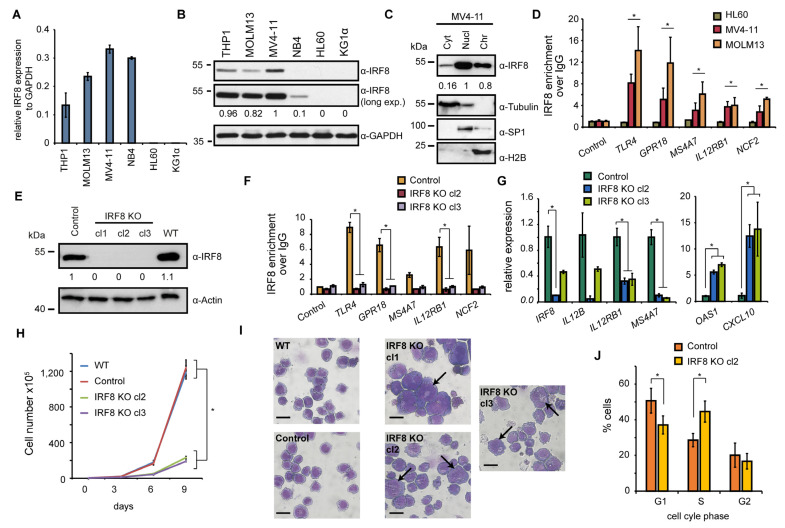
IRF8 deletion impairs cellular function of MV4-11 cells. (**A**) Gene expression of IRF8 relative to GAPDH in several AML cell lines, measured by RT-qPCR (*n* = 2). (**B**) Western blot of IRF8 in AML cell lines. (**C**) Cellular distribution of IRF8 in MV4-11 cells. Tubulin, SP1, and H2B were used as controls for cytoplasm (Cyt), nucleoplasm (Nucl), and chromatin-bound fractions (Chr), respectively. (**D**) ChIP-qPCR experiments at several IRF8 target genes in HL60, MV4-11, and MOLM13 cells. Significance was calculated by comparing IRF8 targets versus the control genomic region (*n* = 2). (**E**) Western blot of IRF8 knockout clones in MV4-11 cells, compared to wildtype (WT) and cells infected with a non-targeting CRISPR construct (Control). (**F**) ChIP-qPCR at IRF8 target genes using IRF8 antibody in IRF8 KO cells compared to control cells (*n* = 2). (**G**) Gene expression changes of interferon response genes upon IRF8 deletion in MV4-11 cells (*n* = 2). (**H**) Cell counting of wildtype, CRISPR control and IRF8 knockout cells (*n* = 2). (**I**) Pappenheim staining of IRF8 knockout cells in comparison to control cells. Arrows indicate enlarge cells. Scale = 10 µm. (**J**) Cell cycle analysis of IRF8 knockout cell (clone 2) in comparison to control cells (*n* = 2). * *p* < 0.05 (Student’s *t*-test). The uncropped blots are shown in Figure S1.

To investigate the role of IRF8 in IRF8-dependent AML cancer cells in more detail, we selected six AML cell lines (THP1, MOLM13, MV4-11, NB4, HL60, and KG1α) and analyzed the expression of IRF8 in those cells. HL60 and KG1α cells have not been investigated yet in a CRISPR screen [45], thus it is unknown whether the cells may be affected by IRF8 deletion. The other four cell lines were included in the CRISPR screen and three of them (THP1, MOLM13, MV4-11) had a strong negative score in this screen (Figure 2E). Via RT-qPCR, we confirmed a strong expression of IRF8 in THP1, MOLM13, and MV4-11 cells (Figure 3A). We additionally found IRF8 expression in NB4 cells, but no expression was detected in KG1α and HL60 cells. By Western blotting we validated that IRF8 is particularly strongly expressed in MV4-11 cells, while in other investigated cell lines IRF8 was either less abundantly expressed or undetectable (Figure 3B). For further investigation we therefore focused on the MV4-11 cell line, which is a commonly studied AML cell line. MV4-11 belongs to the M5 subtype of AML and bears a FLT3-ITD mutation [48], which occurs in about 30% of all AML cases [5]. The high expression of IRF8 in those cells, as well as the strong negative CRISPR score (Figure 2E), suggests that MV4-11 cells are strongly dependent on IRF8 expression and thus represent a suitable model to study the role of IRF8 in IRF8-dependent AML.

To approach the role of IRF8 in MV4-11 cells, we first investigated its cellular localization. We found IRF8 is predominantly present in the nucleus and at the chromatin (Figure 3C), suggesting that the main function of IRF8 in MV4-11 cells is related to gene regulation, consistent with its function as a transcription factor [8]. Using chromatin immunoprecipitation (ChIP), we could confirm that IRF8 is enriched at several interferon response target genes, such as TLR4 and IL12RB1, in MV4-11 and MOLM13 cells, but not in HL-60 cells, where IRF8 is not expressed (Figure 3D). This result demonstrates the enrichment of IRF8 at individual genes, which supports the role of IRF8 for gene regulation in IRF8 expressing AML cells. Since no enrichment was observed in HL-60 cells, high specificity of the IRF8 antibody in ChIP experiments is ascertained.

To gain insights into the gene regulatory role of IRF8, we created IRF8 knockout cells using the CRISPR/Cas9 system, whereby IRF8 was completely deleted. The successful targeting of the IRF8 locus and the absence of IRF8 protein has been confirmed by Western blotting (Figure 3E). Additionally, we could confirm that IRF8 was not present at its target genes anymore by ChIP-qPCR experiments (Figure 3F), further demonstrating the successful knockout of IRF8 in those cells. An investigation of genes known to be affected by IRF8 perturbation, such as OAS1, CXCL10, IL12B, and IL12RB1 [49,50,51], confirmed a strong change of their expression upon IRF8 deletion. Some of these genes, including OAS1, become robustly upregulated, while others get downregulated (Figure 3G), suggesting that the absence of IRF8 substantially influences the transcriptional program of the MV4-11 cells.

Upon examination of the MV4-11 cells, we observed a strongly impaired proliferation of the IRF8 knockout cells compared to the control cells (Figure 3H). We detected an about 1.5-fold increase of the doubling time (from 25 h to 35 h), which is consistent with the negative score in the CRISPR screen after IRF8 deletion (Figure 2E). Closer inspection of the cells revealed that the knockout cells show a distinct morphology, compared to wildtype and CRISPR control cells. In particular, we observed that the knockout cells are generally more heterogenous, have often enlarged nuclei, and their surface is more irregular compared to the wildtype cells (Figure 3I). Besides the morphological changes, we detected elevated numbers of dead cells and alterations of the cell cycle, with an increase in the S phase, and a decrease of the G1 and G2 phase (Figure 3J), which may indicate a block in cell cycle progression, leading to reduced cell growth.

Together these results suggest that the function of the cells is massively compromised upon IRF8 deletion, both at the transcriptional and cellular level. Given the transcriptional function of IRF8, we hypothesized that IRF8 possibly influences the cellular properties by modulating the gene regulatory network.

### 3.4. IRF8 Targets Ten-Thousands of Genomic Loci in MV4-11 Cells

**Figure 4:**
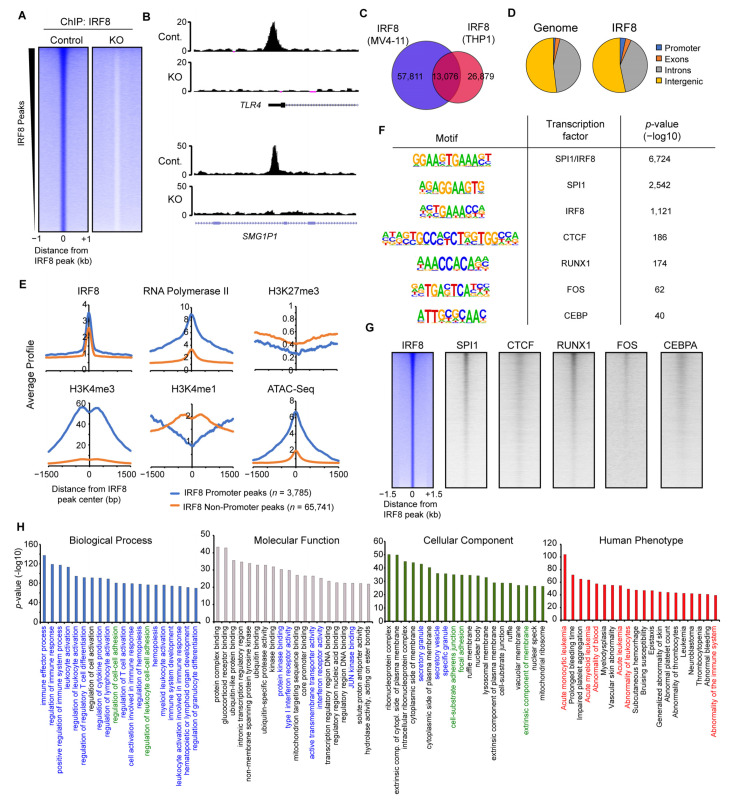
IRF8 binds to thousands of genomic loci in MV4-11 cells. (**A**) Heatmap of significant IRF8 peaks in Control and IRF8 knockout cells. (**B**) Genome browser view of two IRF8 peaks, at the *TLR4* promoter and at an enhancer of *SMG1P1.* (**C**) Venn diagram showing the overlap of IRF8 ChIP-Seq results from MV4-11 cells and THP1 cells [29]. (**D**) Genomic distribution of IRF8 in MV4-11 cells, in comparison to the genome. (**E**) Histone modifications and RNA-Polymerase II at promoter or non-promoter IRF8 peaks. (**F**) Enriched motifs identified at IRF8 peaks. (**G**) Heatmaps showing the comparison of IRF8 with other transcription factors. (**H**) Gene ontologies associated with IRF8 target loci, identified using Genomic Regions Enrichment of Annotations Tool (GREAT) [33]. Colorized ontologies are related to immune response pathways (blue), cell adhesion pathways (green) and disease ontologies associated with leukemia (red).

To elaborate the mechanism of how IRF8 modulates the function of AML cells, we next addressed the genomic targets of IRF8 in MV4-11 cells and their role in gene regulation. Via ChIP-seq experiments, we identified almost 70,000 locations with bound IRF8 (Figure 4A). Those peaks are absent in the IRF8 knockout cells (Figure 4A,B). We compared our IRF8 ChIP-Seq data with the results of the only other previously published IRF8 ChIP-Seq experiment in human cells, performed in the previously mentioned AML cell line THP1 [29]. The analysis identified a substantial overlap between these two independent experiments (Figure 4C), suggesting that IRF8 is distributed similar yet distinct in MV4-11 and THP1 cell lines.

Investigation of the IRF8 binding pattern within the MV4-11 genome (Figure 4D) revealed that IRF8 is slightly enriched at promoter regions, but it is mostly present at non-promoter sites, indicating that IRF8 predominantly targets enhancers in the genome. Fittingly, analyses of histone modifications and RNA-Polymerase II showed that the IRF8-bound promoter sites are enriched for H3K4me3 and RNA-Polymerase II, while the IRF8-targeted non-promoter sites are enriched for the enhancer mark H3K4me1 (Figure 4E). IRF8 bound locations are generally depleted of the repressive H3K27me3 mark, suggesting that these IRF8 bound loci are in an active and accessible state, which allows the binding of IRF8 to the DNA. This conclusion is supported by ATAC-Seq data, which shows that IRF8-targeted locations have increased accessibility (Figure 4E).

Motif analysis demonstrated that the IRF8 peaks are most strongly enriched for a motif that is a composite of the ETS and IRF transcription factor binding motifs (Figure 4F), consistent with the known cooperation between IRF8 and the ETS transcription factor SPI1 (PU.1) [52]. Besides motifs for SPI1, several other transcription factor-binding motifs are enriched at IRF8-bound locations, such as those for RUNX1, FOS, CTCF, and CEBP. We confirmed the colocalization of IRF8 with those transcription factors, using publicly available ChIP-Seq datasets from AML cells (Figure 4G) [29,37,38,39,40]. These results suggest that IRF8 works in concert with additional transcription factors such as SPI1, RUNX1 and others to influence the gene transcription in MV4-11 cells.

To identify the biological functions of the genes targeted by IRF8, we made use of GREAT (Genomic Regions Enrichment of Annotations Tool) [33], which links the annotated regulatory region to their cognate gene targets. Applying the identified IRF8-bound loci to GREAT we found many gene ontologies highly significantly enriched. Consistent with the known function of IRF8 in the immune system [49], the IRF8-bound loci are mainly involved in immune response regulation and related pathways (Figure 4H). This strong association is additionally supported by the cellular components, which outline an enrichment for secretory granules and vesicles. However, besides immune response related gene ontologies, other features such as cell–cell adhesion, ribonucleoprotein complexes, nuclear bodies, ubiquitination-, and transcription-associated ontologies, are also enriched (Figure 4H), suggesting that IRF8-bound loci are involved in regulating many cellular functions in MV4-11 cells that are not directly linked to the immune response. Intriguingly, in the “Human Phenotype” gene ontology category [53], acute monocytic and myeloid leukemia are top terms associated with the IRF8-targeted loci. This suggests that IRF8-mediated gene regulation in MV4-11 cells contributes to the regulatory processes that are involved in AML onset, which is in line with our principal hypothesis.

Together these investigations demonstrate that IRF8 binds to tens of thousands of loci in the genome of AML cells, which are associated with critical biological pathways, implicating that IRF8 is an important transcriptional regulator in MV4-11 cells.

### 3.5. IRF8 Regulates Key Signaling Molecules

**Figure 5:**
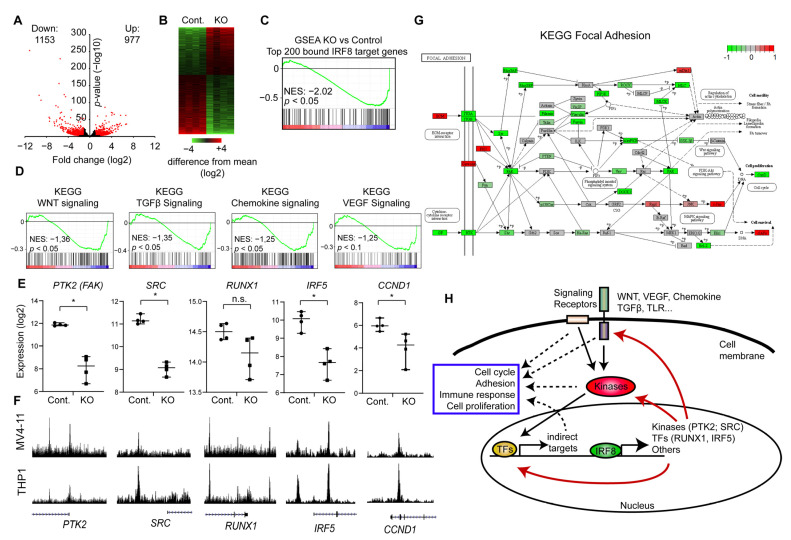
IRF8 deletion downregulates key signaling and focal adhesion pathways. (**A**) Volcano-plot showing up- and downregulated genes upon IRF8 deletion. (**B**) Heatmap depicting the up and down regulated genes. (**C**) Gene set enrichment analysis (GSEA) of the top 200 genes with the highest IRF8 promoter levels, demonstrating that those genes become predominantly downregulated upon IRF8 deletion. NES = Normalized Enrichment Score (**D**) GSEA of several important signaling pathways, that become downregulated upon IRF8 deletion. NES = Normalized Enrichment Score. (**E**) Gene expression changes of key regulators upon IRF8 deletion, based on RNA-Seq data. * *p* < 0.05, n.s. not significant. (Student’s *t*-test). (**F**) Enrichment of IRF8 at the genes from (**E**) in MV4-11 (this study) and THP1 cells [29]. (**G**) Kyoto Encyclopedia of Genes and Genomes (KEGG)-pathway analysis of focal adhesion. Green are components that are downregulated upon IRF8 deletion, while red boxes indicate upregulated factors. (**H**) Model of the function of IRF8 in AML cells.

To investigate the impact of IRF8 on the transcription and the biological processes in AML cells, we performed RNA-Seq in IRF8 knockout and CRISPR Control MV4-11 cells, in four replicates. The data were mapped to the human transcriptome and subsequently the differentially expressed genes were identified. Consistent with the strong phenotype, we identified more than 2000 significantly differentially expressed genes (*p* < 0.01, fold change >2) (Figure 5A,B). A roughly equal number of up- and downregulated genes were detected, which supports that IRF8 can act both as repressor and activator, as described previously [54]. However, using GSEA (Gene Set Enrichment Analysis) we found the top 200 genes with the highest IRF8 promoter levels are significantly downregulated upon IRF8 deletion (Figure 5C), suggesting that, in this context, IRF8 functions predominantly as an activator.

To understand how IRF8 deletion influences the properties of the cell, we used our RNA-Seq data and performed GSEA [43], with a focus on KEGG (Kyoto Encyclopedia of Genes and Genomes) pathways. Unexpectedly, we found that many signaling pathways are downregulated, which are not typically described to be influenced by IRF8. Specifically, we found that the Wnt, Chemokine, TGFβ, and VEGF signaling pathway are significantly downregulated in the IRF8 deleted cells (Figure 5D), suggesting that the absence of IRF8 impairs the normal signaling process of the cells. Closer inspection showed, that IRF8 absence strongly reduces the expression of critical kinases, including the SRC kinase and the focal adhesion kinase (FAK, PTK2) (Figure 5E). Importantly, investigation of the respective genes, showed that the IRF8 protein directly binds to those genes in the genome (Figure 5F), implicating that IRF8 is involved in their transcriptional regulation in MV4-11 cells. An enrichment of IRF8 can also be observed in the previously published IRF8 ChIP-Seq experiment in THP1 cells [29], suggesting that IRF8-mediated regulation of these genes is not restricted to just one cell line. In addition, we found that IRF8 binds and modulates several critical transcriptional regulators, such as RUNX1 and IRF5, which are downstream effectors of many signaling pathways [47,55], implicating a global and sophisticated regulatory function of IRF8 in AML cells. Given that RUNX binding motif are found at IRF8 binding sites (Figure 4F), it is likely that RUNX1 additionally cooperates with IRF8 for gene regulation, which could lead to a positive feedback loop.

Both SRC and FAK are regulators of focal adhesion [56], which directly affects the cellular properties and proliferation of immune cells [57]. Thus, we investigated the consequences for focal adhesion in more detail. KEGG analysis revealed that the focal adhesion pathway is indeed strongly negatively affected upon IRF8 knockout, as many components of this pathway are downregulated (Figure 5G). This includes the Protein Kinase B (PKB/AKT) and PAK7, which are associated with poor prognosis in AML [58,59]. We further observe a significantly reduced expression of the downstream effector Cyclin D1 (CCND1) (Figure E,G) [60], which is also a direct target of IRF8 (Figure 5E,F). Cyclin D1 is often upregulated in cancer and a master regulator of G1 phase progression [61]. Its downregulation upon IRF8 knockout may in part contribute to the reduced proliferation observed upon IRF8 knockout (Figure 3H). Focal adhesions are further important to AML development by enhancing the attachment of the AML cells to the bone marrow niche, which increases the resistance to chemotherapeutic drugs [62]. Thus, in regulating focal adhesions, high IRF8 expression may contribute to the occurrence of relapse and poorer prognosis of AML patients (Figure 2B) [63].

Collectively, our results suggest that IRF8 is critical for enhancing the gene transcription of essential signaling molecules and transcriptional regulators in AML cells, and that the absence of IRF8 leads to an impaired expression of pro-tumorigenic factors involved in cell signaling, focal adhesion and cell cycle (Figure 5H).

## 4. Discussion

In this study, we aimed to identify novel AML-specific susceptibility genes. In our approach, we used three distinct criteria to select potential candidates: CRISPR perturbation score from AML cell lines, overexpression in AML patients’ samples, and association with poor prognosis in patients. This approach is similar but distinct from another recently published study [64]. In Zhou et al., CRISPR perturbation data have also been explored, but instead of overexpression and patients’ survival, the druggability of the factor was used as an additional criterium. Consequently, this previous study did not pick up IRF8 as a potential target in AML, likely because no drug for the inhibition of IRF8 has been established yet. Nonetheless, given that our strategy identified several well-known AML susceptibility genes, such as RUNX1, FLT3, and SPI1 (PU.1) [6,47,48], the validity of our approach is demonstrated. In addition, BCL2 (B-cell lymphoma 2), which has been identified in our analysis (Figure 1D), but not in that of Zhou et al., has recently been proposed to be a suitable drug target of AML patients [65]. Thus, these two distinct approaches lead to complementary lists of candidates, which could be a valuable source to select candidates for future studies. Besides IRF8, which this study investigated in more detail, several other interesting and novel candidates were identified by our selection process. IKZF1 (Ikaros) is a transcription factor that plays important gene regulatory roles, particularly in the cell fate decision of lymphoid cells [66]. In the context of AML, some studies suggest that it is occasionally deleted or mutated in AML [67,68]. However, recent genome-wide analysis demonstrated that IKZF1 is highly expressed in most AML samples [46]. Furthermore, the deletion of IKZF1 leads to reduced proliferation of AML cells [45], suggesting that AML depend on high IKZF1 expression. We also observed an association of high IKZF1 expression and a poorer prognosis for AML patients (Figure 1D). Thus, IKZF1 could play a pro-tumorigenic function in AML, which awaits further investigation. Another interesting transcription factor is MEF2D, which is often rearranged in ALL development [69]. Its overexpression in AML may contribute to the AML proliferation, similar to IRF8. Further analysis of the role of these two transcription factors may show, that these proteins could be suitable drug targets, as well. Notably, ZEB2 and GFI1 have been found to be important for AML in several studies [70,71], but our approach has excluded both proteins as candidates, as they do not correlate with poor prognosis, at least when using the data from TCGA (Figure 1D). Future research will be required to determine whether patient’s survival data are suitable to narrow down relevant candidates or whether it will possibly exclude appropriate targets.

To substantiate the findings of our in silico analysis by in vivo experiments, we characterize the role of one of the candidates in more detail. We selected IRF8, given that the related protein IRF5 can chemically be inhibited [23], suggesting that IRF8 could possibly be targeted in the future, as well. IRF8 has previously been described as a tumor suppressor [17,18], while our data suggest an oncogenic function in AML. Thus, we speculated that IRF8 could have an atypical role in AML. Our in vivo work could indeed confirm that IRF8 deletion strongly impairs the proliferation of the AML cell line MV4-11, which shows robust IRF8 expression in WT cells. This finding is consistent with the CRISPR screen, showing that cells with high IRF8 expression strongly require IRF8 for efficient proliferation (Figure 2E). Our work further demonstrated that IRF8 plays a genome-wide gene regulatory role, by regulating thousands of genes (Figure 4). Most of those IRF8-associated genes are linked to immune response or cellular adhesion, but also to many other important cellular processes, such as transcription (Figure 4H). Consistently, gene expression data showed that upon IRF8 deletion many genes are mis-regulated, including the key transcription factors RUNX1 and IRF5, which control many aspects of cellular processes [47,55] (Figure 5E).

As consequences, many members of key signaling pathways showed diminished expression, potentially explaining the impaired proliferation upon IRF8 deletion (Figure 5H). In particular, the reduced expression of members of the Wnt, Chemokine, and TGFβ signaling pathway are possibly critical for this effect, given that those pathways control many aspects of cell homeostasis [72,73], and have been proposed to contribute to cancer development [74,75,76] including AML [77,78,79]. Additionally, we observed that the central regulators of focal adhesion, namely the focal adhesion kinase FAK (PTK2) and its downstream effector Cyclin D1, showed massively reduced expression in the IRF8 knockout cells (Figure 5E,G,H), which possibly contributes to the proliferation defect, as well [62,63]. However, we investigated the IRF8 knockout cells after they have reached a steady state. It is therefore likely that many of the observed effects on transcription and the cellular properties are secondary consequences of the IRF8 deletion (Figure 5H). Thus, it would be interesting to assess the most important down-stream effectors of IRF8 in AML.

Although our work supports a pro-oncogenic role of IRF8 in AML, it has several limitations. The initial analysis uses public datasets that are either based on cell lines, or on a limited number of patients. Thus, it would be important to further validate the role of IRF8 in human AML with primary cells or patient derived xenograft models. Moreover, the TCGA dataset, where we identified a poorer prognosis for high IRF8 expressing AMLs (Figure 2C), is rather small, and lacks important information, such as the AML subtype. Thus, the usage of larger datasets that include AML subtypes would be critical to assess whether AML-types differ in IRF8 expression and dependency. Given that the AML cell lines that are affected by IRF8 KO in the CRISPR screens were generated form patients suffering from distinct AML types (Table S3), it however suggests that IRF8-dependent AML can occur in different AML classes. Furthermore, investigations on whether certain treatments are particularly effective in IRF8-high expressing AMLs, as proposed for tamibarotene [20], could increase knowledge about the role IRF8 in AML, and its usability as biomarker.

Since our cell-based experiments were mainly performed in the MV4-11 cell line, which shows a very high IRF8 expression, the conclusions about the molecular role of IRF8 in AML is restricted. Although IRF8 has an overall similar chromatin binding pattern in THP1 and MV4-11 cells (Figure 4C), it may differ in other cell lines. Thus, it is unknown whether the consequences of IRF8 deletion are similar or different in various AML cell lines, and whether the consequences may depend on the specific AML type, or AML-related mutations. Therefore, an expansion of this initial study in more AML cell lines or primary cells would be important to better understand the molecular mechanisms how IRF8 contribute to AML cell proliferation.

Interestingly, previous studies proposed that IRF8 acts as a tumor suppressor in some leukemia types, while our analyses strongly support an oncogenic function of IRF8 in human AML. Therefore, the question arises how these seemingly contradictory roles of IRF8 in distinct leukemia types can be explained. Most studies that describe a tumor-suppressor role of IRF8 in leukemia refer to CML [14,15,17,80,81,82] or the AML M3 subclass APL [18,19]. IRF8 function could differ between various leukemia types. Consistently, in the CRISPR screen only the AML cells, but not other leukemia cells, showed strongly reduced proliferation upon IRF8 deletion (Figure 2C). Several possibilities may explain the contrasting role of IRF8 in miscellaneous leukemias. For example, IRF8 may interact with distinct cofactors in AML and non-AML leukemia, which may alter its transcriptional activity, and consequently its impact on transcription. Further, IRF8 may cooperate with other transcription factors that are also highly expressed in AML to regulate gene transcription. For example, we showed that the IRF8 binding pattern in MV4-11 cells strongly overlaps with RUNX1 or PU.1 (Figure 4G), which are known oncogenes in AML [6,47]. In other cancer types, the transcriptional set-up may be different, which may lead to an alternative genomic distribution of IRF8, and thereby to a disparate role in gene regulation. To our knowledge, no ChIP-Seq data of IRF8 in human non-AML cell lines exists. Therefore, more work is required to better understand the opposing role of IRF8 in AML and non-AML leukemia cells.

Contradictory data can be found for the role of IRF8 in AML. In an early study, low IRF8 expression was detected both in AML and CML patients [16], but more recent genome-wide data suggest that IRF8 is rather highly expressed in AML (Figure 2A,B). High IRF8 expression has been proposed to be an adverse prognostic factor for patients with AML [83], supporting the negative role of IRF8. On the other hand, a study in mouse suggests that high IRF8 expression inhibits AML activation [84]. This work has been done in the context of MN1-induced AML, and it remains unclear whether this finding can be translated to human AML. This study also found that high IRF8 expression in the AML cell line OCI-AML3 leads to reduced tumor size in xenograft experiments, supporting a tumor-suppressor function of IRF8 in those cells. In contrast, the OCI-AML3 cells score strongly negative in CRISPR screens (Figure 2E), suggesting that IRF8 is required for the proliferation of these cells. The overexpression of IRF8 in a cell line that already has high IRF8 levels (Figure 2D) may lead to a dosage dependent effect and could be toxic for the cells. Thus, further work would be important to clarify, whether IRF8 acts predominantly as oncogene or as tumor-suppressor in human AML. Nonetheless, our bioinformatical and cellular investigations strongly support a pro-tumorigenic role of IRF8 in a subset of AML cells, but not in other leukemia cells (Figure 2C). Thus, we propose that IRF8 could be a suitable molecular target, specifically in AML.

Recent work suggests that also transcription factors [7], including interferon regulatory factors [23], can potentially be targeted by small molecule inhibitors. The development of inhibitors for transcription factors is typically more challenging compared to enzyme inhibitors, given that no enzymatic pocket exists that could easily be targeted. Additional efforts will thus be required to obtain functional small molecules that are able to target IRF8. We also inspected other IRFs and related factors in the CRISPR screen data. Besides IRF8, only the protein IRF2BP2, a co-repressor of IRF2, has a strong negative score in AML cell lines (Table S2). This finding suggests that within the IRF protein family, IRF8 has a special function in AML, which cannot be fulfilled by the other IRFs. Thus, in the future, it will be of great interest to test whether selective IRF8 inhibition can perturb the cell proliferation of AML cells, and whether those inhibitors are suitable to treat AML patients. Previous attempts to optimize the treatment of AML patients, such as the use of kinase inhibitors [85], has so far not dramatically improved the survival rate of AML patients [1]. The development of small-molecule inhibitors, that target alternative molecules, such as IRF8, could establish an improved treatment for AML patients.

## 5. Conclusions

In summary, our investigation of publicly available data revealed novel factors to be specifically essential in AML. We validated the critical role of IRF8 in a human AML cell line and demonstrated that IRF8 is involved in the regulation of multiple genes that are important for pro-proliferative settings in AML cells. Thus, IRF8 could possibly serve as a molecular marker and might be a suitable drug target for the treatment of AML.

## Data Availability

ChIP-Seq and RNA-Seq data have been deposited to the Gene Expression Omnibus (GEO) with accession number GSE163275.

## Supplementary Materials

The following are available online at https://www.mdpi.com/2072-6694/13/4/764/s1, Figure S1: Full Western blots, Table S1: RT-qPCR primers, Table S2: Average CRISPR Score for each gene in AML and other tumors cells (related to Figure 1B), Table S3: FAB classification of cell lines, IRF8 expression and CRISPR score.

## References

[1] De Kouchkovsky I., Abdul-Hay M. (2016). Acute myeloid leukemia: A comprehensive review and 2016 update. Blood Cancer J..

[2] Kuykendall A., Duployez N., Boissel N., Lancet J.E., Welch J.S. (2018). Acute Myeloid Leukemia: The Good, the Bad, and the Ugly. Am. Soc. Clin. Oncol. Educ. Book.

[3] Patel J.P., Gonen M., Figueroa M.E., Fernandez H., Sun Z., Racevskis J., Van Vlierberghe P., Dolgalev I., Thomas S., Aminova O. (2012). Prognostic relevance of integrated genetic profiling in acute myeloid leukemia. N. Engl. J. Med..

[4] DiNardo C.D., Cortes J.E. (2016). Mutations in AML: Prognostic and therapeutic implications. Hematol. Am. Soc. Hematol. Educ. Program.

[5] Daver N., Schlenk R.F., Russell N.H., Levis M.J. (2019). Targeting FLT3 mutations in AML: Review of current knowledge and evidence. Leukemia.

[6] Takei H., Kobayashi S.S. (2019). Targeting transcription factors in acute myeloid leukemia. Int. J. Hematol..

[7] Chen A., Koehler A.N. (2020). Transcription Factor Inhibition: Lessons Learned and Emerging Targets. Trends Mol. Med..

[8] Honda K., Takaoka A., Taniguchi T. (2006). Type I interferon [corrected] gene induction by the interferon regulatory factor family of transcription factors. Immunity.

[9] Salem S., Salem D., Gros P. (2010). Role of IRF8 in immune cells functions, protection against infections, and susceptibility to inflammatory diseases. Hum. Genet..

[10] Becker A.M., Michael D.G., Satpathy A.T., Sciammas R., Singh H., Bhattacharya D. (2012). IRF-8 extinguishes neutrophil production and promotes dendritic cell lineage commitment in both myeloid and lymphoid mouse progenitors. Blood.

[11] Yanez A., Ng M.Y., Hassanzadeh-Kiabi N., Goodridge H.S. (2015). IRF8 acts in lineage-committed rather than oligopotent progenitors to control neutrophil vs monocyte production. Blood.

[12] Lee K.Y., Geng H., Ng K.M., Yu J., van Hasselt A., Cao Y., Zeng Y.X., Wong A.H., Wang X., Ying J. (2008). Epigenetic disruption of interferon-gamma response through silencing the tumor suppressor interferon regulatory factor 8 in nasopharyngeal, esophageal and multiple other carcinomas. Oncogene.

[13] Luo X., Xiong X., Shao Q., Xiang T., Li L., Yin X., Li X., Tao Q., Ren G. (2017). The tumor suppressor interferon regulatory factor 8 inhibits beta-catenin signaling in breast cancers, but is frequently silenced by promoter methylation. Oncotarget.

[14] Scheller M., Schonheit J., Zimmermann K., Leser U., Rosenbauer F., Leutz A. (2013). Cross talk between Wnt/beta-catenin and Irf8 in leukemia progression and drug resistance. J. Exp. Med..

[15] Holtschke T., Lohler J., Kanno Y., Fehr T., Giese N., Rosenbauer F., Lou J., Knobeloch K.P., Gabriele L., Waring J.F. (1996). Immunodeficiency and chronic myelogenous leukemia-like syndrome in mice with a targeted mutation of the ICSBP gene. Cell.

[16] Schmidt M., Nagel S., Proba J., Thiede C., Ritter M., Waring J.F., Rosenbauer F., Huhn D., Wittig B., Horak I. (1998). Lack of interferon consensus sequence binding protein (ICSBP) transcripts in human myeloid leukemias. Blood.

[17] Burchert A., Cai D., Hofbauer L.C., Samuelsson M.K., Slater E.P., Duyster J., Ritter M., Hochhaus A., Muller R., Eilers M. (2004). Interferon consensus sequence binding protein (ICSBP; IRF-8) antagonizes BCR/ABL and down-regulates bcl-2. Blood.

[18] Gaillard C., Surianarayanan S., Bentley T., Warr M.R., Fitch B., Geng H., Passegue E., de The H., Kogan S.C. (2018). Identification of IRF8 as a potent tumor suppressor in murine acute promyelocytic leukemia. Blood Adv..

[19] Liu X., Chen J., Yu S., Yan L., Guo H., Dai J., Zhang W., Zhu J. (2017). All-trans retinoic acid and arsenic trioxide fail to derepress the monocytic differentiation driver Irf8 in acute promyelocytic leukemia cells. Cell Death Dis..

[20] McKeown M.R., Johannessen L., Lee E., Fiore C., di Tomaso E. (2019). Antitumor synergy with SY-1425, a selective RARalpha agonist, and hypomethylating agents in retinoic acid receptor pathway activated models of acute myeloid leukemia. Haematologica.

[21] Pelish H.E., Liau B.B., Nitulescu I.I., Tangpeerachaikul A., Poss Z.C., Da Silva D.H., Caruso B.T., Arefolov A., Fadeyi O., Christie A.L. (2015). Mediator kinase inhibition further activates super-enhancer-associated genes in AML. Nature.

[22] McKeown M.R., Corces M.R., Eaton M.L., Fiore C., Lee E., Lopez J.T., Chen M.W., Smith D., Chan S.M., Koenig J.L. (2017). Superenhancer Analysis Defines Novel Epigenomic Subtypes of Non-APL AML, Including an RARalpha Dependency Targetable by SY-1425, a Potent and Selective RARalpha Agonist. Cancer Discov..

[23] Song S., De S., Nelson V., Chopra S., LaPan M., Kampta K., Sun S., He M., Thompson C.D., Li D. (2020). Inhibition of IRF5 hyperactivation protects from lupus onset and severity. J. Clin. Investig..

[24] Thompson C.D., Matta B., Barnes B.J. (2018). Therapeutic Targeting of IRFs: Pathway-Dependence or Structure-Based?. Front. Immunol..

[25] Sanjana N.E., Shalem O., Zhang F. (2014). Improved vectors and genome-wide libraries for CRISPR screening. Nat. Methods.

[26] Volkel S., Stielow B., Finkernagel F., Stiewe T., Nist A., Suske G. (2015). Zinc finger independent genome-wide binding of Sp2 potentiates recruitment of histone-fold protein Nf-y distinguishing it from Sp1 and Sp3. PLoS Genet.

[27] Langmead B., Trapnell C., Pop M., Salzberg S.L. (2009). Ultrafast and memory-efficient alignment of short DNA sequences to the human genome. Genome Biol..

[28] Zhang Y., Liu T., Meyer C.A., Eeckhoute J., Johnson D.S., Bernstein B.E., Nusbaum C., Myers R.M., Brown M., Li W. (2008). Model-based analysis of ChIP-Seq (MACS). Genome Biol..

[29] Mohaghegh N., Bray D., Keenan J., Penvose A., Andrilenas K.K., Ramlall V., Siggers T. (2019). NextPBM: A platform to study cell-specific transcription factor binding and cooperativity. Nucleic Acids Res..

[30] Liu T., Ortiz J.A., Taing L., Meyer C.A., Lee B., Zhang Y., Shin H., Wong S.S., Ma J., Lei Y. (2011). Cistrome: An integrative platform for transcriptional regulation studies. Genome Biol..

[31] Ramirez F., Dundar F., Diehl S., Gruning B.A., Manke T. (2014). deepTools: A flexible platform for exploring deep-sequencing data. Nucleic Acids Res..

[32] Heinz S., Benner C., Spann N., Bertolino E., Lin Y.C., Laslo P., Cheng J.X., Murre C., Singh H., Glass C.K. (2010). Simple combinations of lineage-determining transcription factors prime cis-regulatory elements required for macrophage and B cell identities. Mol. Cell.

[33] McLean C.Y., Bristor D., Hiller M., Clarke S.L., Schaar B.T., Lowe C.B., Wenger A.M., Bejerano G. (2010). GREAT improves functional interpretation of cis-regulatory regions. Nat. Biotechnol..

[34] Kent W.J., Sugnet C.W., Furey T.S., Roskin K.M., Pringle T.H., Zahler A.M., Haussler D. (2002). The human genome browser at UCSC. Genome Res..

[35] Gerlach D., Tontsch-Grunt U., Baum A., Popow J., Scharn D., Hofmann M.H., Engelhardt H., Kaya O., Beck J., Schweifer N. (2018). The novel BET bromodomain inhibitor BI 894999 represses super-enhancer-associated transcription and synergizes with CDK9 inhibition in AML. Oncogene.

[36] Gollner S., Oellerich T., Agrawal-Singh S., Schenk T., Klein H.U., Rohde C., Pabst C., Sauer T., Lerdrup M., Tavor S. (2017). Loss of the histone methyltransferase EZH2 induces resistance to multiple drugs in acute myeloid leukemia. Nat. Med..

[37] Phanstiel D.H., Van Bortle K., Spacek D., Hess G.T., Shamim M.S., Machol I., Love M.I., Aiden E.L., Bassik M.C., Snyder M.P. (2017). Static and Dynamic DNA Loops form AP-1-Bound Activation Hubs during Macrophage Development. Mol. Cell.

[38] Pott S., Kamrani N.K., Bourque G., Pettersson S., Liu E.T. (2012). PPARG binding landscapes in macrophages suggest a genome-wide contribution of PU.1 to divergent PPARG binding in human and mouse. PLoS ONE.

[39] Prange K.H.M., Mandoli A., Kuznetsova T., Wang S.Y., Sotoca A.M., Marneth A.E., van der Reijden B.A., Stunnenberg H.G., Martens J.H.A. (2017). MLL-AF9 and MLL-AF4 oncofusion proteins bind a distinct enhancer repertoire and target the RUNX1 program in 11q23 acute myeloid leukemia. Oncogene.

[40] Heinz S., Texari L., Hayes M.G.B., Urbanowski M., Chang M.W., Givarkes N., Rialdi A., White K.M., Albrecht R.A., Pache L. (2018). Transcription Elongation Can Affect Genome 3D Structure. Cell.

[41] Dobin A., Davis C.A., Schlesinger F., Drenkow J., Zaleski C., Jha S., Batut P., Chaisson M., Gingeras T.R. (2013). STAR: Ultrafast universal RNA-seq aligner. Bioinformatics.

[42] Love M.I., Huber W., Anders S. (2014). Moderated estimation of fold change and dispersion for RNA-seq data with DESeq2. Genome Biol..

[43] Subramanian A., Tamayo P., Mootha V.K., Mukherjee S., Ebert B.L., Gillette M.A., Paulovich A., Pomeroy S.L., Golub T.R., Lander E.S. (2005). Gene set enrichment analysis: A knowledge-based approach for interpreting genome-wide expression profiles. Proc. Natl. Acad. Sci. USA.

[44] Luo W., Brouwer C. (2013). Pathview: An R/Bioconductor package for pathway-based data integration and visualization. Bioinformatics.

[45] Meyers R.M., Bryan J.G., McFarland J.M., Weir B.A., Sizemore A.E., Xu H., Dharia N.V., Montgomery P.G., Cowley G.S., Pantel S. (2017). Computational correction of copy number effect improves specificity of CRISPR-Cas9 essentiality screens in cancer cells. Nat. Genet..

[46] Tang Z., Li C., Kang B., Gao G., Zhang Z. (2017). GEPIA: A web server for cancer and normal gene expression profiling and interactive analyses. Nucleic Acids Res..

[47] Sood R., Kamikubo Y., Liu P. (2017). Role of RUNX1 in hematological malignancies. Blood.

[48] Quentmeier H., Reinhardt J., Zaborski M., Drexler H.G. (2003). FLT3 mutations in acute myeloid leukemia cell lines. Leukemia.

[49] Shin D.M., Lee C.H., Morse H.C. (2011). IRF8 governs expression of genes involved in innate and adaptive immunity in human and mouse germinal center B cells. PLoS ONE.

[50] Langlais D., Barreiro L.B., Gros P. (2016). The macrophage IRF8/IRF1 regulome is required for protection against infections and is associated with chronic inflammation. J. Exp. Med..

[51] Yamamoto M., Kato T., Hotta C., Nishiyama A., Kurotaki D., Yoshinari M., Takami M., Ichino M., Nakazawa M., Matsuyama T. (2011). Shared and distinct functions of the transcription factors IRF4 and IRF8 in myeloid cell development. PLoS ONE.

[52] Huang W., Horvath E., Eklund E.A. (2007). PU.1, interferon regulatory factor (IRF) 2, and the interferon consensus sequence-binding protein (ICSBP/IRF8) cooperate to activate NF1 transcription in differentiating myeloid cells. J. Biol. Chem..

[53] Kohler S., Gargano M., Matentzoglu N., Carmody L.C., Lewis-Smith D., Vasilevsky N.A., Danis D., Balagura G., Baynam G., Brower A.M. (2021). The Human Phenotype Ontology in 2021. Nucleic Acids Res..

[54] Tamura T., Kurotaki D., Koizumi S. (2015). Regulation of myelopoiesis by the transcription factor IRF8. Int. J. Hematol..

[55] Takaoka A., Yanai H., Kondo S., Duncan G., Negishi H., Mizutani T., Kano S., Honda K., Ohba Y., Mak T.W. (2005). Integral role of IRF-5 in the gene induction programme activated by Toll-like receptors. Nature.

[56] Huveneers S., Danen E.H. (2009). Adhesion signaling—Crosstalk between integrins, Src and Rho. J. Cell Sci..

[57] Harjunpaa H., Llort Asens M., Guenther C., Fagerholm S.C. (2019). Cell Adhesion Molecules and Their Roles and Regulation in the Immune and Tumor Microenvironment. Front. Immunol..

[58] Martelli A.M., Nyakern M., Tabellini G., Bortul R., Tazzari P.L., Evangelisti C., Cocco L. (2006). Phosphoinositide 3-kinase/Akt signaling pathway and its therapeutical implications for human acute myeloid leukemia. Leukemia.

[59] Quan L., Cheng Z., Dai Y., Jiao Y., Shi J., Fu L. (2020). Prognostic significance of PAK family kinases in acute myeloid leukemia. Cancer Gene Ther..

[60] Zhao J., Pestell R., Guan J.L. (2001). Transcriptional activation of cyclin D1 promoter by FAK contributes to cell cycle progression. Mol. Biol. Cell.

[61] Musgrove E.A., Caldon C.E., Barraclough J., Stone A., Sutherland R.L. (2011). Cyclin D as a therapeutic target in cancer. Nat. Rev. Cancer.

[62] Gruszka A.M., Valli D., Restelli C., Alcalay M. (2019). Adhesion Deregulation in Acute Myeloid Leukaemia. Cells.

[63] Recher C., Ysebaert L., Beyne-Rauzy O., Mansat-De Mas V., Ruidavets J.B., Cariven P., Demur C., Payrastre B., Laurent G., Racaud-Sultan C. (2004). Expression of focal adhesion kinase in acute myeloid leukemia is associated with enhanced blast migration, increased cellularity, and poor prognosis. Cancer Res..

[64] Zhou Y., Takacs G.P., Lamba J.K., Vulpe C., Cogle C.R. (2020). Functional Dependency Analysis Identifies Potential Druggable Targets in Acute Myeloid Leukemia. Cancers.

[65] Konopleva M., Pollyea D.A., Potluri J., Chyla B., Hogdal L., Busman T., McKeegan E., Salem A.H., Zhu M., Ricker J.L. (2016). Efficacy and Biological Correlates of Response in a Phase II Study of Venetoclax Monotherapy in Patients with Acute Myelogenous Leukemia. Cancer Discov..

[66] Georgopoulos K. (2017). The making of a lymphocyte: The choice among disparate cell fates and the IKAROS enigma. Genes Dev..

[67] de Rooij J.D., Beuling E., van den Heuvel-Eibrink M.M., Obulkasim A., Baruchel A., Trka J., Reinhardt D., Sonneveld E., Gibson B.E., Pieters R. (2015). Recurrent deletions of IKZF1 in pediatric acute myeloid leukemia. Haematologica.

[68] Zhang X., Li X., Lv Y., Zhu Y., Wang J., Jin J., Yu W. (2020). The specific distribution pattern of IKZF1 mutation in acute myeloid leukemia. J. Hematol. Oncol..

[69] Gu Z., Churchman M., Roberts K., Li Y., Liu Y., Harvey R.C., McCastlain K., Reshmi S.C., Payne-Turner D., Iacobucci I. (2016). Genomic analyses identify recurrent MEF2D fusions in acute lymphoblastic leukaemia. Nat. Commun..

[70] Li H., Mar B.G., Zhang H., Puram R.V., Vazquez F., Weir B.A., Hahn W.C., Ebert B., Pellman D. (2017). The EMT regulator ZEB2 is a novel dependency of human and murine acute myeloid leukemia. Blood.

[71] Volpe G., Walton D.S., Grainger D.E., Ward C., Cauchy P., Blakemore D., Coleman D.J.L., Cockerill P.N., Garcia P., Frampton J. (2017). Prognostic significance of high GFI1 expression in AML of normal karyotype and its association with a FLT3-ITD signature. Sci. Rep..

[72] Clevers H., Nusse R. (2012). Wnt/beta-catenin signaling and disease. Cell.

[73] Zhang Y., Alexander P.B., Wang X.F. (2017). TGF-beta Family Signaling in the Control of Cell Proliferation and Survival. Cold Spring Harb. Perspect. Biol..

[74] Zhan T., Rindtorff N., Boutros M. (2017). Wnt signaling in cancer. Oncogene.

[75] Ikushima H., Miyazono K. (2010). TGFbeta signalling: A complex web in cancer progression. Nat. Rev. Cancer.

[76] Chow M.T., Luster A.D. (2014). Chemokines in cancer. Cancer Immunol. Res..

[77] Kittang A.O., Hatfield K., Sand K., Reikvam H., Bruserud O. (2010). The chemokine network in acute myelogenous leukemia: Molecular mechanisms involved in leukemogenesis and therapeutic implications. Curr. Top. Microbiol. Immunol..

[78] Gruszka A.M., Valli D., Alcalay M. (2019). Wnt Signalling in Acute Myeloid Leukaemia. Cells.

[79] Dong M., Blobe G.C. (2006). Role of transforming growth factor-beta in hematologic malignancies. Blood.

[80] Nardi V., Naveiras O., Azam M., Daley G.Q. (2009). ICSBP-mediated immune protection against BCR-ABL-induced leukemia requires the CCL6 and CCL9 chemokines. Blood.

[81] Deng M., Daley G.Q. (2001). Expression of interferon consensus sequence binding protein induces potent immunity against BCR/ABL-induced leukemia. Blood.

[82] Hao S.X., Ren R. (2000). Expression of interferon consensus sequence binding protein (ICSBP) is downregulated in Bcr-Abl-induced murine chronic myelogenous leukemia-like disease, and forced coexpression of ICSBP inhibits Bcr-Abl-induced myeloproliferative disorder. Mol. Cell. Biol..

[83] Pogosova-Agadjanyan E.L., Kopecky K.J., Ostronoff F., Appelbaum F.R., Godwin J., Lee H., List A.F., May J.J., Oehler V.G., Petersdorf S. (2013). The prognostic significance of IRF8 transcripts in adult patients with acute myeloid leukemia. PLoS ONE.

[84] Sharma A., Yun H., Jyotsana N., Chaturvedi A., Schwarzer A., Yung E., Lai C.K., Kuchenbauer F., Argiropoulos B., Gorlich K. (2015). Constitutive IRF8 expression inhibits AML by activation of repressed immune response signaling. Leukemia.

[85] Fernandez S., Desplat V., Villacreces A., Guitart A.V., Milpied N., Pigneux A., Vigon I., Pasquet J.M., Dumas P.Y. (2019). Targeting Tyrosine Kinases in Acute Myeloid Leukemia: Why, Who and How?. Int. J. Mol. Sci..

