# Supplementary figures and images for: IRF8 Is an AML-Specific Susceptibility Factor That Regulates Signaling Pathways and Proliferation of AML Cells
Source: Cancers (Basel). 2021 Feb 12;13(4):764. doi: 10.3390/cancers13040764 (PMC7917770; doi:10.3390/cancers13040764)

Figure 3 B

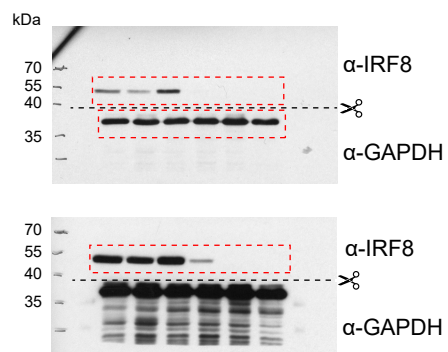

Figure 3C

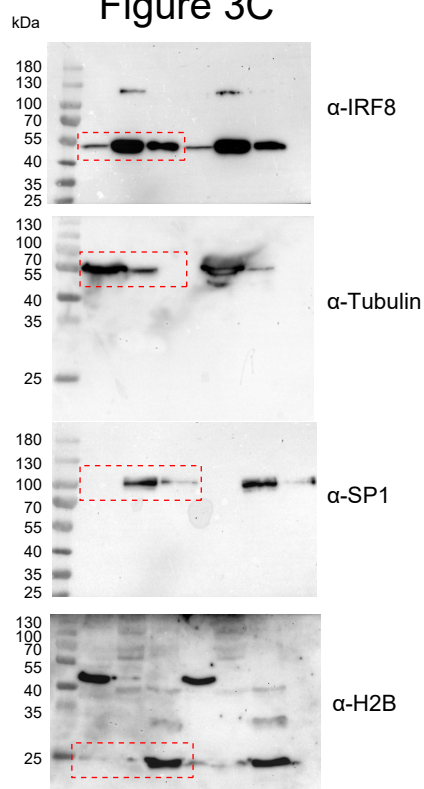

Figure 3E

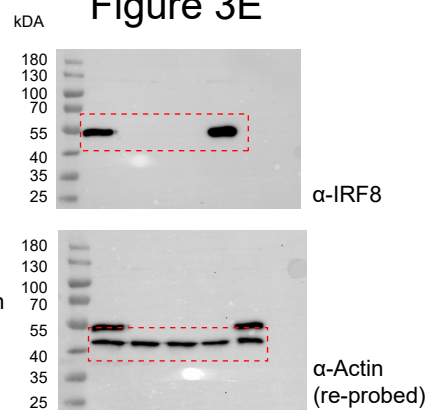

Supplement: Supplementary file 1 [file cancers-13-00764-s001.zip › SuppFigure1.pdf]
